# Clinical correlation of metabolic parameters on ^18^F-FDG PET/CT in idiopathic frozen shoulder

**DOI:** 10.1007/s12149-016-1147-y

**Published:** 2017-02-06

**Authors:** Kyoung Sook Won, Du Hwan Kim, Duk Hyun Sung, Bong-Il Song, Hae Won Kim, Kwang-Soon Song, Si-Wook Lee, Chul-Hyun Cho

**Affiliations:** 1 Department of Orthopedic Surgery, Pain Research Center, Dongsan Medical Center, School of Medicine, Keimyung University, 56 Dalseong-ro, Jung-gu, Daegu, 41931 South Korea; 2 Department of Nuclear Medicine, Dongsan Medical Center, School of Medicine, Keimyung University, 56 Dalseong-ro, Jung-gu, Daegu, 700-712 South Korea; 3 Department of Rehabilitation Medicine, Dongsan Medical Center, School of Medicine, Keimyung University, 56 Dalseong-ro, Jung-gu, Daegu, 700-712 South Korea; 4 Department of Physical and Rehabilitation Medicine, Samsung Medical Center, School of Medicine, Sungkyunkwan University, Seoul, South Korea

**Keywords:** Frozen shoulder, Pathophysiology, ^18^F-FDG PET/CT, Axillary recess, Rotator interval

## Abstract

**Objective:**

Because positron emission tomography/computed tomography (PET/CT) using fluorine-18-fluorodeoxyglucose (^18^F-FDG) can be used to visualize inflammation of the musculoskeletal system, it may help elucidate the pathophysiology of frozen shoulder (FS). The purpose of this study was to characterize the uptake pattern on ^18^F-FDG PET/CT in patients with idiopathic FS and to determine if there is a correlation between its metabolic parameters and clinical findings.

**Methods:**

^18^F-FDG PET/CT was conducted to 35 patients with unilateral idiopathic FS. Clinical data including pain, functional scores, and passive range of motion (ROM) were collected. Maximum standardized uptake values (SUVmax) were measured at the four regions of interest (ROIs): rotator interval (RI), anterior joint capsule (AJC), axillary recess (AR), and posterior joint capsule (PJC) from the attenuation-corrected axial images.

**Results:**

Mean SUVmax values for four ROIs of the affected shoulder were significantly higher than those of the unaffected shoulder. Mean SUVmax values of RI and AR were significantly higher than those of AJC and PJC and mean SUVmax of AJC was significantly higher than that of PJC in the affected side. Three recognizable patterns of increased uptake were noted: (1) AR dominant type (15 patients); (2) RI dominant type (9 patients); (3) both RI and AR dominant type (11 patients). The SUVmax of AR showed negative correlation with abduction and forward flexion. The SUVmax of RI showed negative correlation with external rotation and internal rotation. The SUVmax of AJC showed negative correlation with all ROMs. However, there was no significant correlation between the SUVmax of PJC and any ROM.

**Conclusion:**

Our study demonstrates that the anterior–inferior capsular portion, including RI and AR, is the main pathologic site of idiopathic FS and reveals significant correlations between ROM and metabolic parameters on ^18^F-FDG PET/CT. These results imply that AR and RI lesions are related to elevational limitations and rotational limitations, respectively.

## Introduction

Frozen shoulder (FS) is one of the most common shoulder disorders which is characterized by shoulder pain and limited range of motion (ROM) [1]. FS is thought to be the result of inflammation in the joint synovium triggered by unknown factors and then a gradual onset of fibrosis [2, 3]. However, the exact pathophysiology of idiopathic FS remains debatable.

Arthroscopy and imaging studies have demonstrated loss of axillary recess (AR) fold, capsular contracture, rotator interval (RI) thickening and fibrosis including contracture of the coracohumeral ligament, and adhesion of the subacromial bursa in FS [3–7]. Clarifying the pathologic site may affect treatment methods (e.g., optimizing injection sites or the extent of capsular release). Although magnetic resonance image (MRI) is useful when diagnosing shoulder pain, it has high rate of false positives [4, 8]. Recent studies demonstrated that specific patterns in positron emission tomography (PET) using fluorine-18-fluorodeoxyglucose (^18^F-FDG) are related to shoulder diseases including osteoarthritis and FS [9–12]. Because positron emission tomography/computed tomography (PET/CT) using ^18^F-FDG can visualize inflammation of the musculoskeletal system and provide a comparison of both sides, we expected that PET/CT would supply new information about the pathophysiology of FS.

Although the diagnostic extent or patterns of ROM in FS have not been standardized, it is well accepted that FS has ROM limitation in all directions. Cyriax [13] proposed that tightness in a joint capsule would result in a specific pattern of proportional motion restriction. However, most shoulder specialists have felt that there is heterogeneity of ROM limitation in FS. Some studies also reported that no single capsular pattern emerges in FS [14, 15]. To date, there are few reports that explain the relationship between the pathologic sites and patterns of ROM limitation.

The purpose of this study was to determine whether there are specific patterns or outstanding sites of ^18^F-FDG uptake of the shoulder joint on ^18^F-FDG PET/CT for idiopathic FS and to elucidate which areas of uptake are related to the patterns of ROM limitation. We hypothesized that ^18^F-FDG uptake would be focused in the AR and RI, and uptake in AR would be related to elevational limitations and uptake in RI would be related to rotational limitations.

## Materials and methods

### Study subjects

This prospective study was conducted from February to July 2015 in patients who were diagnosed as having unilateral idiopathic FS. The ethics committee of our institution approved this study. The purpose of the study and the possibility of radio-hazard were explained to all patients. The patients were willing to participate in this study and provided written informed consent. We performed ^18^F-FDG PET/CT without charge for a total of 35 patients enrolled. Standard and meticulous history taking and physical examination to differentiate idiopathic FS from other shoulder disorders were evaluated. Inclusion criteria included: shoulder pain with limitation of passive ROM of greater than 30° in two or more planes of movement; stage II or III defined by Hannafin and Chiaia [16]. Plain radiography, MRI and/or diagnostic ultrasonography were also routinely used to exclude other shoulder problems including symptomatic rotator cuff tears, calcific tendinitis, and osteoarthritis. Exclusion criteria included: FS secondary to endocrine disorders (diabetes mellitus, thyroid disease); rheumatic diseases; infection; trauma; fracture; previous shoulder or adjacent region surgeries or interventions; cervical radiculopathy; rotator cuff disease; osteoarthritis; history of cancer; previous steroid injection within 3 months. Of the 45 patients eligible for this study, 5 patients were excluded because of bilateral involvement, 4 refused to perform this study, 1 was excluded because of history of breast cancer.

Clinical assessment included visual analog scale (VAS) pain score, American Shoulder and Elbow Surgeons (ASES) score, and Subjective Shoulder Value (SSV). Passive ROMs including forward flexion, abduction, external rotation with arm at the side, and internal rotation at the back were evaluated.

### ^18^F-Fluorodeoxyglucose positron emission tomography/computed tomography

All patients fasted for at least 6 h before the PET/CT. Blood glucose levels were measured and were lower than 150 mg/dL in all patients. PET/CT was performed using a Biograph mCT PET/CT scanner (Siemens Healthcare). Regional CT of shoulders was performed with a continuous spiral technique on a 64-slice helical CT scanner (CARE dose 4D and CARE KV on quality reference mAs 55 and reference KV 120, respectively; pitch 1.0; section width 3 mm) in the supine position with the arms down. Next, an emission scan was performed at 90 s per bed at 60 min after the intravenous administration of 3.5 MBq/kg of ^18^F-FDG. CT data were used for attenuation correction and PET images were reconstructed with a three-dimensional (3D) ordered-subsets expectation maximization algorithm (21 subsets, two iterations). CT and PET scan data were coregistered on a dedicated workstation (SingoMMWP VE40A, Siemens Healthcare). ^18^F-FDG PET scan images were displayed on the transaxial, coronal, and sagittal planes, and with rotating 3D images (voxel size = 2.5 × 2.5 × 3.0 mm, transaxial resolution = 4 mm FWHM).

### Image analysis

**Fig. 1:**
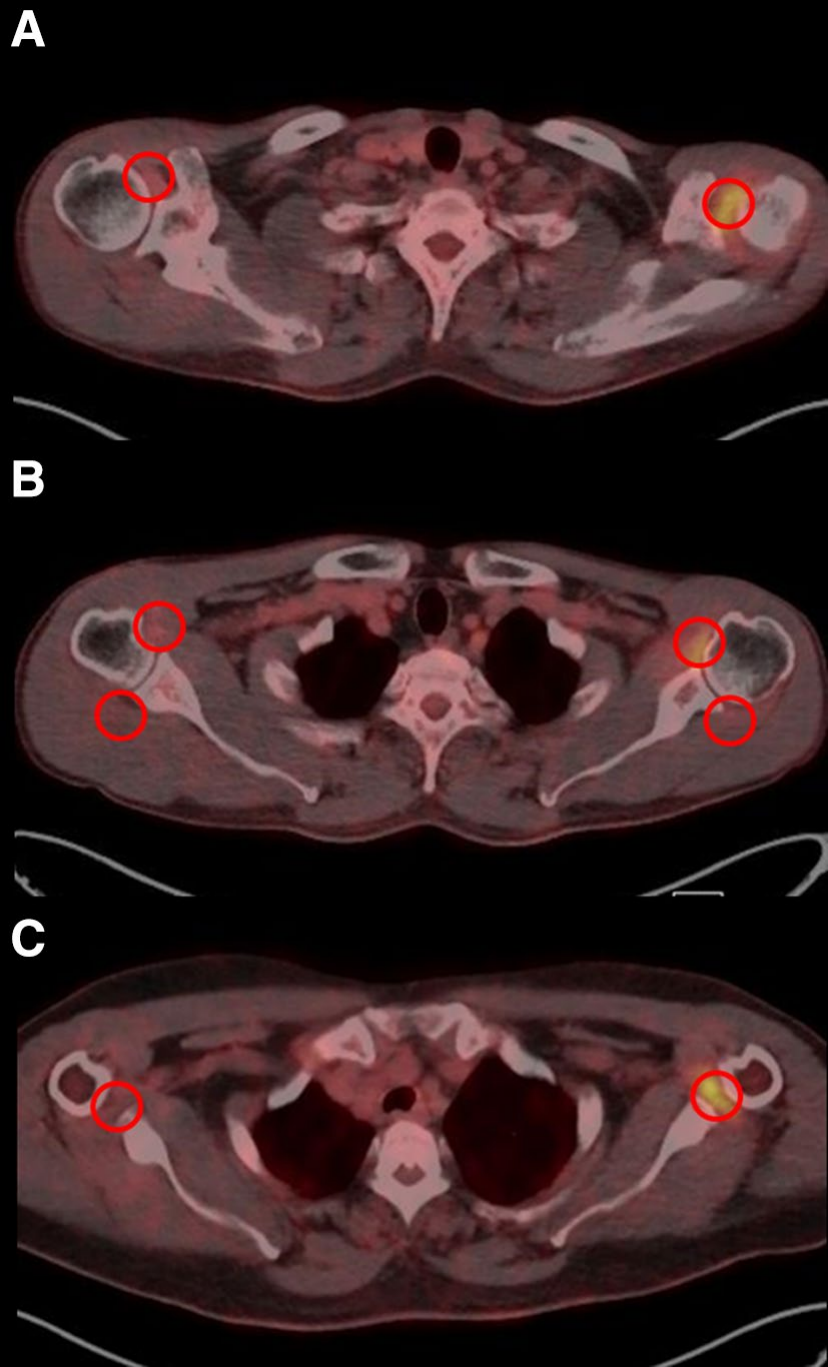
Standardized uptake values measured at the four regions of interest from the attenuation corrected axial images on ^18^F-FDG PET/CT. **a** Rotator interval. **b** Anterior and posterior joint capsule. **c** Axillary recess

An experienced nuclear medicine specialist evaluated qualitatively for location, distribution, and relative intensity of radiotracer uptake in the shoulder joint to identify the outstanding pattern of FS. We evaluated the intensity of ^18^F-FDG accumulation as standardized uptake value (SUV), defined as the tissue concentration divided by the activity injected per body weight. SUVs were measured at the four regions of interest (ROIs): RI, anterior joint capsule (AJC), AR, and posterior joint capsule (PJC) from the attenuation-corrected axial images (Fig. 1). Maximum SUV (SUVmax) at a pixel with highest uptake of ^18^F-FDG within each ROI was recorded. SUVs at the unaffected shoulder were also measured for control group.

### Statistical analysis

MedCalc version 14.12.0 (MedCalc Software bvba, Belgium) was used for analysis. Paired *t* tests were used to compare SUVmax between affected and unaffected shoulders. One-way analysis of variance (ANOVA) was used to compare SUV_max_ of four ROIs in the affected shoulder. If the one-way ANOVA demonstrated statistically significant differences among the four ROIs, multiple comparison result was performed by contrast as Bonferroni correction. For statistical analysis of internal rotation, we converted values into contiguously numbered groups: T1–T12 for 1–12; L1–L5 for 13–17; sacrum to 18; pelvis to 19; and buttock to 20. The correlation between clinical parameters and SUVmax was assessed using the Pearson correlation coefficient. All statistical tests were conducted at the two-sided 5% significance level and all *p* values reported were, correspondingly, two-sided.

## Results

The characteristics of the study population are given in Table 1. The average age was 53.6 years and a greater population was of female gender (65.7%). The average duration of symptoms was 6.1 months. Thirty patients had stage II FS and five had stage III FS.

**Table 1:** Patients’ characteristics

Variables	Values
Age (years)	53.6 ± 7.5
Sex (M:F)	12:23
Duration of symptoms (months)	6.1 ± 3.9
Stage (I:II:III:IV)	0:35:5:0
ASES score	38.7 ± 13.2
SSV (%)	34.8 ± 15.1%
VAS pain score	6.5 ± 1.5
ABD (°)	86.7° ± 21.0°
FF (°)	102.6° ± 21.0°
ER (°)	31.6° ± 13.3°
IR score	17.8 ± 1.9

### Quantitative analysis of ^18^F-FDG PET/CT

Mean SUVmax values for RI, AJC, AR, and GT of the affected shoulder were significantly higher than those of the unaffected shoulder (all *p* < 0.001) (Table 2). The highest SUVmax in the affected shoulder was noted at AR among regional metabolic parameters, followed by RI and AJC. The SUVmax of PJC in affected shoulder was significantly higher than that of unaffected shoulder, although the difference was small. There were significant differences of mean SUVmax among four ROIs in the affected shoulder (*p* < 0.001). These results indicated that the mean SUVmax values of RI and AR were significantly higher than those of AJC and PJC (*p* < 0.001) and the mean SUVmax of AJC was significantly higher than that of PJC (*p* < 0.001).

**Table 2:** Comparison of metabolic parameters

ROIs	SUVmax in affected shoulder	SUVmax in unaffected shoulder	*p* value^a^	ROIs comparison^b^
RI	6.17 ± 2.59	2.19 ± 0.40	<0.001*	RI, AR > AJC > PJC (*p* < 0.001*)
AJC	4.91 ± 1.17	2.15 ± 0.44	<0.001*	
AR	6.85 ± 2.35	1.92 ± 0.37	<0.001*	
PJC	2.15 ± 0.48	1.51 ± 0.26	<0.001*	

^a^Comparison between affected and unaffected side for each ROI

^b^Multiple comparison by contrast for four ROIs in affected side

*Statistically significant

### Visual analysis of ^18^F-FDG PET/CT

**Fig. 2:**
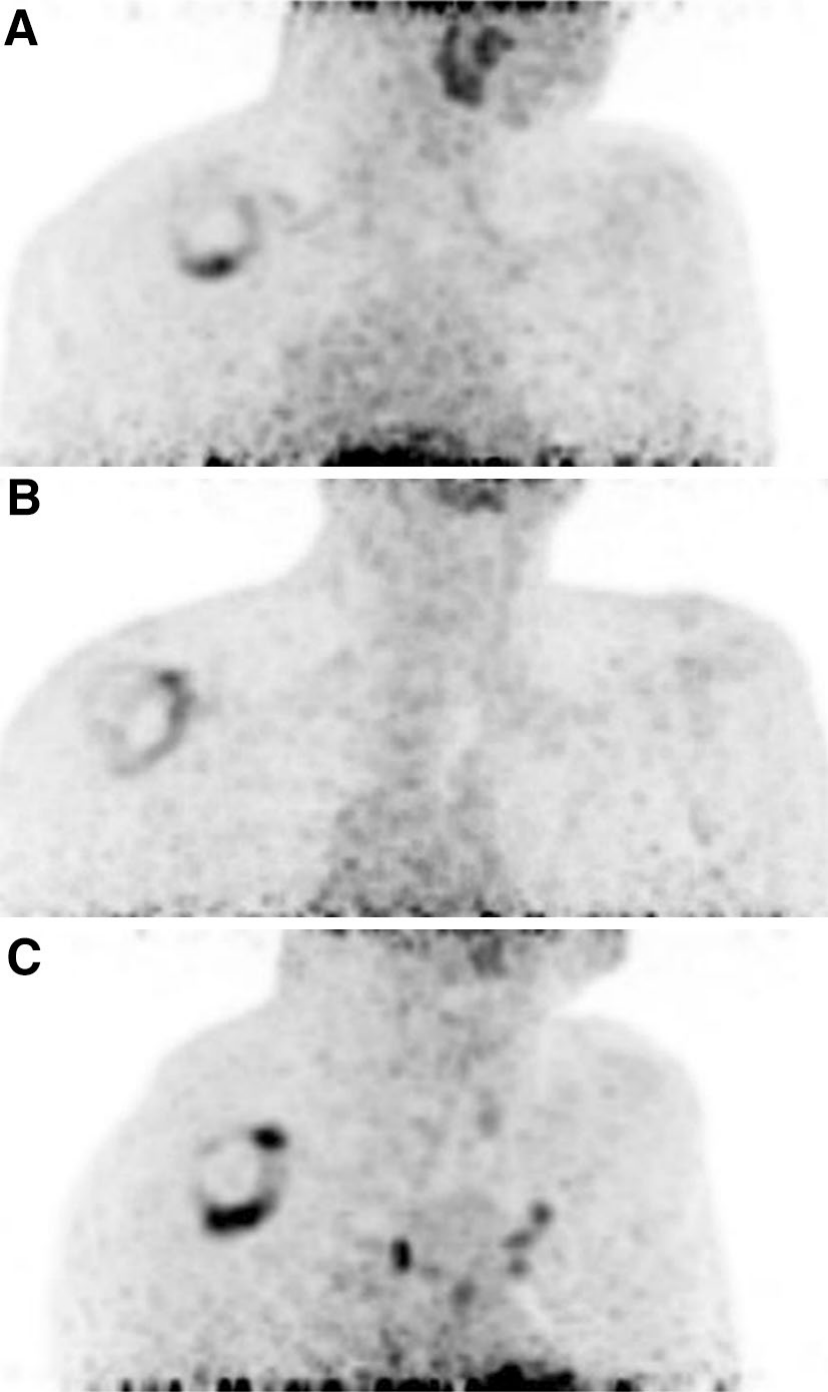
Three recognizable patterns of increased ^18^F-FDG uptake. **a** AR dominant type. **b** RI dominant type. **c** Both RI and AR dominant type

The predominant pattern of ^18^F-FDG uptake in the affected shoulder was increased uptake at AR and/or RI, and spreading into AJC. Three recognizable patterns of increased uptake were noted (Fig. 2): (1) AR dominant type (with or without spreading of the uptake into AJC and or RI); (2) RI dominant type (with or without spreading of the uptake into AJC and/or AR); (3) both RI and AR dominant type with equivalently hot uptake at both RI and AR (with or without spreading of the uptake into AJC). Of the 35 patients with FS, 15 (42.9%) were AR dominant type, 9 (25.7%) were RI dominant type, and 11 (31.4%) were both RI and AR dominant type. There was no increased uptake in the unaffected shoulder.

### Correlation between regional metabolic parameters and range of motion

The SUVmax of RI showed negative correlation with external rotation and internal rotation (*r* = −0.47 and 0.44, *p* = 0.005 and 0.008, respectively). The SUV of AR showed negative correlation with abduction and forward flexion (*r* = −0.46 and −0.46, *p* = 0.006 and 0.005, respectively). The SUV of AJC showed significant correlation with abduction, forward flexion, external rotation and internal rotation (*r* = −0.36, −0.34, −0.52, and 0.50, *p* = 0.033, 0.048, 0.001 and 0.002, respectively). However, there was no significant correlation between the SUV of PJC and ROM (Table 3).

**Table 3:** Correlation between range of motion and regional metabolic parameters

		ABD	FF	ER	IR score
RI—SUVmax	*r*	−0.22	−0.24	−0.47	0.44
	*p* value	0.200	0.157	0.005*	0.008*
AJC—SUVmax	*r*	−0.36	−0.34	−0.52	0.50
	*p* value	0.033*	0.048*	0.001*	0.002*
AR—SUVmax	*r*	−0.46	−0.46	−0.23	0.17
	*p* value	0.006*	0.005*	0.194	0.345
PJC—SUVmax	*r*	−0.10	−0.09	0.01	−0.16
	*p* value	0.578	0.593	0.968	0.365

*Statistically significant

### Correlation between clinical scores and metabolic parameters

There was no significant correlation between clinical scores and metabolic parameters (Table 4).

**Table 4:** Correlation between clinical scores and regional metabolic parameters

		VAS pain score	ASES score	SSV
RI—SUVmax	*r*	0.00	−0.09	0.00
	*p* value	0.995	0.597	0.995
AJC—SUVmax	*r*	−0.05	−0.09	−0.23
	*p* value	0.764	0.597	0.186
AR—SUVmax	*r*	0.04	−0.16	−0.05
	*p* value	0.817	0.401	0.792
PJC—SUVmax	*r*	0.12	−0.15	−0.10
	*p* value	0.493	0.701	0.556

## Discussion

We evaluated cross-sectional ^18^F-FDG PET/CT findings in patients with idiopathic FS who had relatively homogenous characteristics. All patients had significant increases of ^18^F-FDG uptake at the AR, RI, AJC and PJC of the affected shoulder compared to the unaffected shoulder. However, the areas with mean SUVmax >2.5 are AR, RI, and AJC. These results suggest that the main pathologic sites of FS are the AR, RI, and AJC. We also found that the areas of ^18^F-FDG uptake and the patterns of ROM limitation are closely related. The SUVmax of AR showed negative correlation with abduction and forward flexion. The SUVmax of RI showed negative correlation with external rotation and internal rotation. The SUVmax of AJC showed negative correlation with all ROMs. However, there was no significant correlation between the SUVmax of PJC and any ROM.

The main pathologic sites of FS are known to be the coracohumeral ligament and anterosuperior and inferior glenohumeral joint synovium and capsule [1, 17–19]. This knowledge has been confirmed by arthroscopic and MRI findings. Previous arthroscopic findings in refractory FS demonstrated inflamed synovium of the RI region with thickened capsule or hyalinization in the RI capsule and the coracohumeral ligament [17]. Using dynamic MRI with intravenous gadolinum administration, Tamai et al. [19] illustrated an increased perfusion of gadolinum from the vessel to the synovium or capsule of axillary region, which is thought to be the result of synovial and/or capsular inflammation. Our PET/CT study supports previous results suggesting the pathologic sites of FS; however, increased uptake on both RI and AR was not observed in all patients. In fact, only 11 patients (31%) showed increased uptake at both the sites. This finding suggests that the pathologic process of RI and AR do not always occur simultaneously, although this study is a cross-sectional observation. The involvement of RI and AR can be simultaneous or sequential or separate. Mean SUVmax values of AR and RI were significantly higher than those of AJC. No patients had a greater SUVmax in AJC than AR or RI. It is unclear why the SUVmax of AJC was lower than AR or RI. AJC may be a secondary pathologic site following inflammation in RI or AR. Although the differences in SUVmax of PJC were statistically significant between the affected and unaffected shoulders, it may be the result of spreading inflammation since the mean SUVmax of PJC at the affected shoulder was low and the differences between shoulders were minimal. Further research is necessary to clarify synovial geographic differences of the molecular basis in the pathogenesis of FS.

Codman [20] described restricted external rotation movement as one of the features of FS. Clinical investigations confirmed that the contracture of the coracohumeral ligament restricted external rotation movement which is characteristic pathology in FS [21, 22]. Therefore, release of the coracohumeral ligament and thickening capsular release are believed to be the gold standard in the treatment of refractory FS. However, there has been a controversy regarding the extent of capsular release. Some authors suggest that the extent of inferior glenohumeral ligament release should be minimized to prevent damage to the axillary nerve [23, 24], but others reported that global or additive posterior capsular release enhanced ROMs [25, 26]. In the present study, our results suggest that extensive capsular release including posterior capsule is not necessary, but inferior and posterior portions of inferior glenohumeral ligament should be included. Also, individualized capsular release may affect postoperative outcomes. We expect that in patients with an RI dominant type, inferior capsular release will not improve ROM compared to RI and anterior capsular release. In patients with AR or both RI and AR dominant type, the results of anterior and inferior capsular release may be better than anterior capsular release only.

In general, the clinical diagnosis of FS is based on physicians’ physical examination, history taking and negative plain image. There have been clinical efforts to define if consistent patterns or absolute extent of ROM limitation could differentiate FS from other conditions with painful stiffness. The capsular pattern proposed by Cyriax suggests that the external rotation is most limited followed by abduction and then internal rotation [13]. However, following kinematic studies did not support Cyriax’s proposed consistent glenohumeral capsular pattern [14, 15]. Rundquist et al. [14] demonstrated that with the arm abducted, internal rotation was the most limited motion in most patients. The present study suggested that inconsistent patterns of ROM limitation in FS might be related to different areas of capsular inflammation and subsequent fibrosis. Several reports explained the relative contributions of specific portions of the capsule in limiting rotation in cadavers [27, 28]. For example, the whole inferior glenohumeral ligament resists internal rotation from 70° to 90° abduction and the superior glenohumeral ligament resists external rotation at 0° abduction [27, 28]. However, our results in patients with FS were somewhat different from previous studies using cadavers. The SUVmax of AR was related to abduction and forward flexion and not external rotation, while the SUVmax of RI was related to external rotation and internal rotation. Considering difference between ours and cadaveric studies, we have three possibilities to consider. First, the restriction of shoulder motion in patients with FS may be affected by factors other than capsular components. Pain during movement in specific directions may cause restriction in these directions, similar to the cadaveric studies. Second, PET/CT does not reflect the fibrotic contraction of joint capsule, but rather indirectly shows inflammation of the joint capsule or synovium. Increased uptake in a specific area may not guarantee to develop fibrosis. Third, RI including coracohumeral ligament and superior glenohumeral ligament can resist internal rotation as well as external rotation and AR including inferior glenohumeral ligament may also limit forward flexion and abduction movement. We believe that these findings will help determine the most effective site of corticosteroid injection for the treatment of FS. Additional research is needed to evaluate the effect of corticosteroid injection sites according to uptake patterns or patterns of ROM limitation. Another finding of the present study is that metabolic parameters did not correlate with clinical scores including VAS pain score, ASES score, and SSV. Although SUVmax values measured at the four ROIs correlated with ROMs, they did not affect subjective pain and functional scale of the patients.

There are several limitations in this study. First, the sample size was too small to definitively classify patterns of ^18^F-FDG uptake in FS. Second, this study was conducted as a cross-sectional observational study. Our results do not guarantee that we fully understand the entire pathologic process in patients with FS because we did not evaluate enrolled patients longitudinally. Finally, our most weak point is the problem of radiation hazard. To minimize exposure of radiation, we did our best to take regional images and use low doses of ^18^F-FDG thus ensuring that radiation exposure was negligible. Nevertheless, our study has some strong points. We applied strict inclusion criteria to exclude secondary FS. Systemic metabolic diseases related to FS were also excluded. Patient characteristics were homogeneous in that most patients were stage II according to the Hannafin and Chiaia criteria and the duration of symptoms was relatively short. We think that our results may represent PET/CT features of the freezing phase in FS. To the best of our knowledge, our study is the first to quantitatively evaluate PET/CT in idiopathic FS excluding systemic diseases and to analyze the correlation between ROM limitation and images.

In conclusion, our study demonstrates that anteroinferior capsular portion, including RI and AR is the main pathologic site of idiopathic FS and reveals significant correlation between ROM and metabolic parameters on ^18^F-FDG PET/CT. These results imply that AR and RI lesions are related to elevational limitations and rotational limitations, respectively. These findings may help guide the best strategy for treatment including the most appropriate sites of corticosteroid injection and the extent of capsular release during arthroscopic surgery.
